# Household food insecurity and childhood overweight in Jamaica and Québec: a gender-based analysis

**DOI:** 10.1186/1471-2458-11-199

**Published:** 2011-03-31

**Authors:** Lise Dubois, Damion Francis, Daniel Burnier, Fabiola Tatone-Tokuda, Manon Girard, Georgiana Gordon-Strachan, Kristin Fox, Rainford Wilks

**Affiliations:** 1 Faculty of Medicine, University of Ottawa, Institute of Population Health, 1 Stewart Street, office 303, Ottawa, Ontario, K1N 6N5, Canada; 2 Epidemiology Research Unit- TMRI, University of the West Indies, Mona, Kingston 7, Jamaica; 3 Institute of Population Health, University of Ottawa, 1 Stewart Street, Office 304, Ottawa, Ontario, K1N 6N5, Canada; 4 Faculty of Medical Sciences, Deans Office, University of the West Indies, Mona, Kingston 7, Jamaica; 5 Sir Arthur Lewis Institute of Social Sciences and Economic Studies, University of the West Indies, Mona, Kingston 7, Jamaica

## Abstract

**Background:**

Childhood overweight is not restricted to developed countries: a number of lower- and middle-income countries are struggling with the double burden of underweight and overweight. Another public health problem that concerns both developing and, to a lesser extent, developed countries is food insecurity. This study presents a comparative gender-based analysis of the association between household food insecurity and overweight among 10-to-11-year-old children living in the Canadian province of Québec and in the country of Jamaica.

**Methods:**

Analyses were performed using data from the 2008 round of the Québec Longitudinal Study of Child Development and the Jamaica Youth Risk and Resiliency Behaviour Survey of 2007. Cross-sectional data were obtained from 1190 10-year old children in Québec and 1674 10-11-year-old children in Jamaica. Body mass index was derived using anthropometric measurements and overweight was defined using Cole's age- and sex-specific criteria. Questionnaires were used to collect data on food insecurity. The associations were examined using chi-square tests and multivariate regression models were used to estimate odds ratios (OR) and 95% confidence intervals.

**Results:**

The prevalence of overweight was 26% and 11% (p < 0.001) in the Québec and Jamaican samples, respectively. In Québec, the adjusted odds ratio for being overweight was 3.03 (95% CI: 1.8-5.0) among children living in food-insecure households, in comparison to children living in food-secure households. Furthermore, girls who lived in food-insecure households had odds of 4.99 (95% CI: 2.4-10.5) for being overweight in comparison to girls who lived in food-secure households; no such differences were observed among boys. In Jamaica, children who lived in food-insecure households had significantly lower odds (OR 0.65, 95% CI: 0.4-0.9) for being overweight in comparison to children living in food-secure households. No gender differences were observed in the relationship between food-insecurity and overweight/obesity among Jamaican children.

**Conclusions:**

Public health interventions which aim to stem the epidemic of overweight/obesity should consider gender differences and other family factors associated with overweight/obesity in both developed and developing countries.

## Background

In most countries where data are available, the prevalence of childhood overweight has increased [1] to the point of becoming a major public health problem. Although there is some indication that this epidemic may be leveling-off in certain countries over recent years, this evidence is less apparent in lower-SES groups and does not seem to be the case for Canada and some European and Asian countries [2]. Childhood overweight is also associated with numerous short and long-term physiological and psychosocial negative health consequences in both individuals and populations [3-6]. Some studies purport that the epidemic of overweight is due to an increased consumption of low-nutrient, energy-dense products that are high in sugar and fats [7-11]. Others suggest that weight increases are mainly attributable to physical inactivity [10,11].

The childhood overweight epidemic is not restricted to developed countries: a number of lower- and middle-income countries have been struggling with the double burden of underweight and overweight for some time [12,13]. In urban areas of countries undergoing rapid social and economic change (e.g., China, Mexico, Egypt, and Brazil), the prevalence of overweight among children has reached levels comparable to those in developed countries [14]. In western developed countries, childhood adiposity has, for the most part, been shown to inversely associate with socioeconomic status (SES), according to a systematic review of studies from 1990-2005 [15]; whereas, in developing countries, a greater prevalence of childhood overweight has been observed in higher socioeconomic groups [1]. Some gender differences in this association have been noted, however. A seminal review of 144 published studies on the association between SES and overweight/obesity found a strong inverse relationship particularly among women in developed societies; this relationship was inconsistent for men and children. In developing countries, however, a direct association was observed between SES and obesity among men, women, and children [16,17], although a high prevalence of obesity has been reported even among very poor women in developing countries [18].

Another public health problem that concerns both developing and, to a lesser extent, developed countries is food insecurity. Food insecurity arises when individuals do not have sufficient access to safe and nutritious foods at all times, to sustain active and healthy lives [19]. Some studies have reported a paradoxical positive association between food insecurity and childhood obesity [20-22]. However, there have been some inconsistent findings in this research area, with some studies reporting a negative association [23-25] or completely non-existent association between food insecurity and childhood obesity [26,27].

Thus, this study aims, firstly, to examine whether household food insecurity is significantly related to child overweight/obesity in the Canadian province of Québec (total population of less than 8 million) and in the country of Jamaica (total population less than 3 million) and, secondly, to explore gender differences in the association between food insecurity and overweight/obesity in both polities. This study is part of an ongoing collaboration between the University of the West Indies' Epidemiology Research Unit (based in Kingston, Jamaica) and the Institute of Population Health (University of Ottawa, Canada). The prevalence of childhood overweight/obesity is high in both Canada and Jamaica: 26% of 6-to-11-year-old Canadian children were overweight or obese in 2004 according to the age- and sex-specific criteria developed by the International Obesity Task Force [28]; while, in Jamaica, the prevalence of overweight in 11-to-12-year-old children living in the Kingston Metropolitan area was reported to be 19% (BMI ≥ 85^th ^percentile) in 1998 [29]. Furthermore, data from the Canadian Community Health Survey revealed that approximately 7% of Québec households were food-insecure in 2007-2008 [30]. Data on the prevalence of food insecurity among Jamaican households, however, has yet to be published. For the present study, it was hypothesized that a positive association would be observed between household food insecurity and childhood overweight/obesity in Québec, while a negative association would be observed in Jamaica, independent from other factors potentially associated with child overweight/obesity.

## Methods

### Background on study samples

#### Québec

Analyses were conducted using data from the Québec Longitudinal Study of Child Development (QLSCD), a study conducted by Santé Québec, a division of the Institut de la Statistique du Québec (ISQ) [31]. Approval from the Ministry of Health Ethics Committee and consent from participants were obtained. The QLSCD, established to examine the role of familial and social factors in children's health, cognitive, and behavioural development, followed a representative sample (n = 2103) of children born in 1998, in the province of Québec (approximately 70,000 newborns per year), Canada. To ensure geographic representation and minimize the effect of seasonality, the study randomly selected children born throughout the year in each public health geographic area of the province. A public health geographic area or "health region" refers to a geographic unit defined by the provincial ministry of health. Health regions facilitate public health administration for Canadians. Children and their parents were first seen at 5 months (gestational age adjusted for preterm birth) and at one-year intervals thereafter. Standardized, questionnaire-based face-to-face interviews and self-administered questionnaires, completed by the children's mothers and fathers, were used at each cycle of data-collection. Data pertaining to the child was obtained from the person deemed most knowledgeable about the child, which generally was the mother. Information was also obtained from the child's medical records. Of the 2103 infants included in the first cycle of the study, 1190 children remained in the study 10 years later, in 2008.

#### Jamaica

Jamaican data were drawn from the Jamaica Youth Risk and Resiliency Behaviour Survey 2005, conducted by the University of the West Indies [32]. The main purpose of this cross-sectional survey was to monitor the health status, nutritional habits, and lifestyles of children and young teenagers aged 10-15 years, in a nationally representative sample of Jamaican children currently enrolled in school, and to examine how these variables relate to demographic and socioeconomic factors. The data were obtained from the children using trained interviewers who administered questionnaires which were standardized and validated for use in this population. Most children attended primary or secondary schools regularly. The average daily rate of attendance for primary school children was 78.5% [33,34]. Enrolment records, obtained from the Ministry of Education, Youth and Culture, and school attendance registers from selected schools provided the sample frames used in this study.

A multi-stage random sampling method was employed. The first stage involved the random selection of schools within each region where probability was proportional to size. The second stage entailed randomly selecting children from grades within the required age groups. The number of schools selected and the number of students selected per school were proportional to the total number of children in the required age group per parish and school. In 2003, there were 279,986 children in the 10-to-14-year age group and 250,352 in the 15- to-19-year age group. These combined represented approximately 20% of the Jamaican population [35]. Using information on the rate of tobacco use among youths aged 10 to 14 (19%) [36], a confidence level of 95% and an error of + 2% yielded a required sample size of 2,500 children *(EPI-Info *software obtained from the Centers for Disease Control and Prevention [CDC] website). Based on an expected refusal rate of 10%, the sample size was adjusted to 2,800. For this analysis, 1674 children between ages 10 and 11 years were selected for study.

### Measures

#### Overweight and obesity

In Québec, children's heights and weights were measured at home by a trained interviewer following a standardized protocol using a measuring tape, ruler, and scale [37] when children were 10 years old. In Jamaica, body weight, without shoes and with light clothing, was recorded to the nearest 0.1 kg using a calibrated electronic platform scale. Standing height was recorded to the nearest 0.1 cm using a Leicester portable measuring rod. Measurements were obtained at school by trained interviewers following a standardized study protocol. Body mass index (BMI) was calculated as body weight/height^2 ^(kg/m^2^). Overweight and obesity were similarly defined in Québec and Jamaica according to Cole's criteria, which provide age- and sex-specific cut-off points for overweight and obesity in children between 2 and 18 years of age [38].

#### Food insecurity

Food insecurity was assessed in comparable ways in Québec and Jamaica, and fit within the definitional ambits adopted by both the Food and Agriculture Organization (FAO) and the United States Department of Agriculture (USDA) [19,39,40]. In the Québec sample, data on food insecurity were collected via self-administered questionnaires addressed to mothers when children were 10 years of age. Using a 3-point Likert-type scale (rated "Often true", "Sometimes true", "Never true"), mothers were asked to rate how often their families had experienced each of the three following situations: 1) *We eat the same thing several days in a row because we only have a few different kinds of food on hand, and don't have enough money to buy more*; 2) *We eat less than we should because we don't have enough money for food*; and 3) *We can't provide balanced meals for our children because we can't afford it financially*. Children were classified as living in a food-insecure household if mothers answered "often true" or "sometimes true" to any of the three food insecurity statements, and as living in a food-secure household if mothers answered "never true" to each statement.

In the Jamaican sample, children were interviewed to ascertain the presence or absence of food insecurity as well as the extent to which they were food-insecure using two structured questions. The first dichotomous question was: *1) During a usual week, do you go hungry because there is not enough food in your home? *(rated "Yes" or "No"). Children were categorized as being food-insecure if they answered yes to question 1. They were then asked how often they experienced hunger using a 4-point Likert-type rating scale to categorize the extent of food insecurity: *2) During a usual week, how often do you go hungry because there was not enough food in your home? *(rated "Always", "Most of the time", "Sometimes", "Rarely"). This method of combining responses has been employed in other studies on food-insecurity [41,42]. In both questionnaires, the food-insecurity statements gather information at the household level, which has been demonstrated to have both face validity [43,44] and external validity [45,46].

#### Diet and physical activity

In Québec, food consumption (daily consumption of pastries, fruits, and vegetables) was measured by way of a self-administered Food Frequency Questionnaire (FFQ) completed by the children's mothers when the children were 10 years old. The children's mothers were asked: *In the past week, at home and at school (or school's daycare service), on average, how many times during the week or how many times per day has your child eaten the following foods*. The mothers chose one of the following responses: "none", "one to two times per week", "three to four times per week", "five to six times per week", "one time per day", "twice per day", "three times per day", or "four or more times per day". Daily consumption of pastries included pastries, candies, cookies, chips, and chewing gum containing sugar. Daily consumption of fruits excluded the consumption of fruit drinks or juice. Vegetable consumption included potatoes. Parents reported children's level of physical activity by stating whether their child had a "higher" or "much higher" level of physical activity in comparison to other children, or "same", "lower" or "much lower" level than other children. Children's level of physical activity was reported by the parents at age 6. All other variables included in the present study were completed when the children were 10 years old.

In Jamaica, children provided information about their dietary consumption patterns throughout a usual week (i.e., a week without social events that might affect usual intake). A standard questionnaire was validated for use in similar populations of adolescents [47]. Two questions were asked specifically about fruit consumption: *(1) During a usual week, do you eat fruit such as mango, orange, and pawpaw? *Response categories for this first question were: "yes", "no" and "don't know". If the child's response was "yes", then the interviewer proceeded to the following question: *(2) During a usual week, how many times per week do you usually eat fruit, such as mango, orange, and pawpaw? *Response categories were "<1, 1, 2, 3, 4, 5 or more, and don't know". A similarly structured set of questions was asked about usual weekly and daily consumption of vegetables (which did not include potatoes) and pastries (cake, bulla cake, buns, etc.). Physical activity was assessed using the short form of the International Physical Activity Questionnaire (IPAQ) [48] for leisure time physical activity, and children's level of physical activity was classified as "none or low" or "moderate or high".

#### Family type and socioeconomic status (SES)

In Québec, the SES index was calculated by the Institut de la Statistique du Québec [49] based on methods developed by Willms and Shields (1996) that aggregate annual gross income, education level, and occupational prestige for both parents [50]. SES was less rigorously defined in the Jamaican sample due to the unavailability of data: it was based on household crowding terciles and a geographical index (urban, rural, remote rural) developed by the Statistical Institute of Jamaica (STATIN). These two variables were combined to derive a proxy for SES. Family type categories were two-parent family, blended family, and single-parent family in both the Québec and Jamaican samples.

### Statistical analyses

Statistical analyses were conducted using SAS (version 9.2; SAS Institute; Cary, NC) and Stata (Version 10.1 Stata Corp.). In Québec, data were weighted by a factor based on the inverse of the selection probability, the probability of non-response, and the post-stratification and attrition rates to ensure that the data were longitudinally representative of infants born in 1998, in the province of Québec [51]. The weights used were provided by the Institut de la Statistique du Québec and included corrections for response bias. Thus, the rates obtained through this study are comparable to those from other surveys on the same population that use weighted responses, independent of the distribution of the sample [37]. The Jamaican data were weighted by age, sex, school type, and parish to provide normalized weights using data from STATIN.

Univariate associations were verified by performing a chi-square test on contingency tables. The significance level was set at 5%, and significant independent variables and other putative explanatory variables were included in multivariate analyses. Based on the literature, children's sex, level of physical activity, family type, family SES (by tertile: low, medium, high), and children's daily consumption of fruit, vegetables, and pastries were considered potential confounders. Our final models included food insecurity, SES, and family type.

In the Québec model, adjustments were made for pastry and vegetable consumption (significantly associated in the univariate analysis) and physical activity (due to its integral role in the equation of energy balance). The Jamaican model was adjusted for physical activity (significantly associated in the univariate analysis). Kendall Tau's test for correlation was also employed to ensure that SES and household food insecurity were not highly correlated and could be included in the same model as independent variables. Odds ratio (OR) estimates, with 95% confidence intervals, were computed using logistic regressions. The impact of missing data was assessed by conducting with-and-without analyses, which revealed no effect on the outcomes and results seen.

## Results

Characteristics of the Québec and Jamaican children are presented in Table 1. Approximately one-quarter (26%) of children from Québec and one-tenth (11%) of children from Jamaica were overweight or obese. Food insecurity was reported for 9% of the children in Québec and 26% in Jamaica. A greater proportion of children lived in single-parent families in Jamaica (36%) in comparison to Québec (17%). Fruit and vegetable consumption was higher in Québec than in Jamaica, whereas the difference in physical activity was less marked.

**Table 1:** A comparison of 10-year-old Québec and 10-11-year-old Jamaican study participants

		Québec	Jamaica	
**Characteristic**	**Category**	**% (n)**	**% (n)**	**P-value**

Sex	Girl	52.4 (624)	52.6 (881)	0.6792
	Boy	47.6 (566)	47.4 (793)	

Overweight or obese	No	74	89	<.0001
	Yes	26	11	

Family SES	Tertile 1 (Low)	33.0	41.3	<.0001
	Tertile 2 (Middle)	34.1	29.4	
	Tertile 3 (High)	32.9	29.3	

Household food insecurity	No	90.6	73.6	<.0001
	Yes	9.4	26.4	

Family type	Two-parent family	66.3	34.2	<.0001
	Blended family	17.0	29.7	
	Single-parent family	16.7	36.1	

Pastry consumption	Less than once a day	68.7	64.2	0.1375
	Once to less than twice a day	20.3	23.4	
	Twice a day or more	11.0	12.4	

Fruit consumption	Less than once a day	31.1	41.5	<.0001
	Once to less than twice a day	19.1	31.9	
	Twice a day or more	49.8	26.6	

Vegetable consumption	Less than once a day	27.1	59.0	<.0001
	Once to less than twice a day	18.8	25.3	
	Twice a day or more	57.1	15.7	

Physical activity (Québec)	Same, lower or much lower than other children	67.6	//////////	
	Higher or much higher than other children	32.4	//////////	

Physical activity (Jamaica)	None or low	//////////	51.9	
	Moderate or high	//////////	48.1	

Table 2 presents the prevalence of childhood overweight/obesity in Québec and Jamaica according to selected child, family, and household characteristics. In Québec, when boys and girls were analyzed together, the proportion of overweight/obese children was found to be higher in low-SES families (32.2%), in comparison to medium-SES (23.2%) or high-SES (22.2%) families. The proportion of overweight/obese children in food-insecure households (52.5%) was more than double the proportion in food-secure households (23.1%). A higher proportion of overweight/obesity was also observed in children from single-parent families and children who consumed vegetables less than once a day, in comparison to children living in other types of families and children who consumed vegetables more frequently per day, respectively (see also adjusted odds ratios in Table 3). While these trends were similar in boys and girls, the statistically significant findings indicated in Table 2 appear to be driven by differences observed among girls; no statistically significant differences were observed among boys.

**Table 2:** Frequency of overweight or obesity in Québec and Jamaican children by selected characteristics, by sex and by both sexes combined

		Overweight or obese Québec children			Overweight or obese Jamaican children		
		
Characteristic	Category	Girls	Boys	All	Girls	Boys	All
Sex	Girl	/////////////	/////////////	26.3	/////////////	/////////////	12.6
	Boy	/////////////	/////////////	25.3	/////////////	/////////////	9.2

Family SES	Tertile 1 (Low)	34.5*	29.6	32.2*	8.3	5.3*	6.8*
	Tertile 2 (Medium)	22.8	23.6	23.2	14.9	9.5	12.2
	Tertile 3 (High)	21.6	23.0	22.2	14.2	11.6	13.0

Household food insecurity	No	23.2*	23.1	23.1*	13.7	10.5	12.1*
	Yes	66.0	34.6	52.5	9.8	6.1	7.9

Family type	Two-parent family	21.7*	24.5	23.1*	13.8	10.8	12.5
	Blended family	28.8	27.0	28.1	13.5	8.7	11
	Single-parent family	41.9	27.6	35.2	10.8	8.2	9.5

Pastry consumption	Less than once a day	30.7*	25.6	28.4	13.5	10	11.9
	Once to less than twice a day	26.1	20.5	23.1	10.3	8.7	9.5
	Twice a day or more	10.4	24.9	17.1	9.2	6.7	7.9

Fruit consumption	Less than once a day	34.2	28.4	31.4	14.5	9.3	12
	Once to less than twice a day	26.9	19.1	22.8	10.9	9.9	10.4
	Twice a day or more	24.4	24.4	24.4	11.5	7.7	9.6

Vegetable consumption	Less than once a day	41.9*	31.7	37.3*	13.1	9.1	11.2
	Once to less than twice a day	19.9	22.7	21.2	12.5	9.4	10.9
	Twice a day or more	21.9	21.4	21.6	12.5	9.6	11.1

Physical activity (Québec)	Same, lower or much lower than other children	28.8	26.3	27.6	//////////	//////////	//////////
	Higher or much higher than other children	20.4	23.4	21.8	//////////	//////////	//////////

Physical activity (Jamaica)	None or low	//////////	//////////	//////////	15.0*	9.1	12.5
	Moderate or high	//////////	//////////	//////////	10.1	9.2	9.6

* Statistically significant association between the characteristic and the dependent variables within column (chi-square P ≤ 0.05)

**Table 3:** Adjusted odds ratios (OR and 95% CI) for overweight and obese Québec and Jamaican children by selected characteristics, by sex and by both sexes combined

		Overweight or obese Québec children^#^			Overweight or obese Jamaican children^##^		
		
Characteristic	Category	Girls	Boys	All	Girls	Boys	All
Family SES	Tertile 1 (Low)	1	1	1	1	1	1
	Tertile 2 (Middle)	0.87 (0.5-1.5)	0.92 (0.5-1.6)	0.89 (0.6-1.3)	1.87 (1.0-3.4)*	1.75 (0.8-3.9)	1.83(1.1-3.0)*
	Tertile 3 (High)	0.97 (0.5-1.7)	0.91 (0.5-1.7)	0.95 (0.6-1.4)	1.74 (0.9-3.3)	2.37 (1.2-4.9)*	2.00(1.3-3.2)*

Family type	Two-parent family	1	1	1	1	1	1
	Blended family	0.91 (0.5-1.7)	1.14 (0.6-2.2)	1.0 (0.7-1.6)	1.0 (0.6-1.6)	0.59 (0.3-1.6)	0.82(0.6-1.2)
	Single-parent family	1.82 (0.9-3.4)	1.37 (0.7-2.5)	1.63 (1.0-2.5)*	0.79 (0.4-1.3)	0.69(0.3-1.2)	0.74(0.5-1.1)

Household food insecurity	No	1	1	1	1	1	1
	Yes	4.99 (2.4-10.5)*	1.56 (0.7-3.3)	3.03 (1.8-5.0)*	0.72 (0.4-1.2)	0.58 (0.3-1.3)	0.65(0.4-0.9)*

*Significantly different from the reference category (P ≤ 0.05)

# Models adjusted for consumption of pastry and vegetables and for physical activity

## Models adjusted for physical activity

In Jamaica, childhood overweight/obesity was significantly associated only with family SES and household food insecurity. The proportion of overweight/obese children was higher in high-SES families (13%) in comparison to medium-SES (12.2%) or low-SES (6.8%) families. Similarly, a higher proportion of overweight/obesity was found in children from food-secure households (12.1%) in comparison to those in food-insecure households (7.9%).

Sex-specific analyses yielded several significant associations in both the Québec and Jamaican samples. For girls in Québec, childhood overweight/obesity was significantly associated with family SES, household food insecurity, family type, daily consumption of pastries, and daily consumption of vegetables. For boys, no association was observed between childhood overweight/obesity and any of the child, family, or household variables examined. In Jamaica, when the data were analyzed separately for girls and boys, only level of physical activity showed a significant association with overweight/obesity in girls, and only family SES was associated with overweight/obesity in boys.

Multivariate analyses (Table 3) revealed that children in food-insecure households in Québec had odds of 3.03 (95% CI 1.8-5.0) for being overweight or obese, with odds of 4.99 (95% CI 2.4-10.5) for being overweight or obese specifically in girls (when analyzed by gender) in comparison to children in food-secure households. Single-parenting also increased the odds of being overweight or obese by 63% in Québec children. Family SES was no longer significant in multivariate analyses. In Jamaica, the association with childhood overweight/obesity was reversed: higher SES was associated with increased overweight/obesity, and food insecurity was associated with reduced overweight/obesity. More specifically, Jamaican children in food-insecure households had odds of 0.65 (95% CI 0.4-0.9) for being overweight or obese in comparison to those in food-secure households. Similarly, Jamaican children in middle SES (OR 1.83, 95% CI 1.1-3.0) and high SES (OR 2.00, 95% CI 1.3-3.2) families had increased odds of being overweight or obese in comparison to children in low SES families. When boys and girls were considered separately, only SES remained positively associated with overweight/obesity. In comparison to boys and girls in low SES families, boys from high-SES families had odds of 2.37 (95% CI 1.2-4.9) for being overweight or obese, and girls from middle-SES families had odds of 1.87 (95% CI 1.0-3.4). Odds ratio point estimates for boys from middle-SES families and girls from high-SES families increased but failed to reach statistical significance.

## Discussion

The aims of this study were twofold. First, we sought to determine whether household food insecurity was significantly related to childhood overweight/obesity in Québec and in Jamaica. Second, we explored possible gender differences in these associations.

### Overweight and obesity

Overall we found that the prevalence of overweight/obesity differed considerably between Québec (26%) and Jamaica (11%). Findings from the Québec sample corresponded to estimates for Canada as a whole [28] and were also comparable to estimates from other developed countries, such as the United States [52]. However, the prevalence of overweight/obesity among Jamaican children appeared lower than previously reported for similarly aged children [29]. The estimates obtained in the aforementioned study was, however, confined to the urban area of Kingston; whereas, data for our study were representative of all 14 parishes in Jamaica. The use of universal cut-points for overweight/obesity in both adults and children is somewhat controversial due to differences in race/ethnicity and the effects of maturation and genetics [53-55]. Our findings showed clearly that obesity is a critical health problem in both countries. The large disparity in the prevalence of overweight/obesity observed may be attributable to genetic, psychosocial, economic, dietary, and other environmental or behavioral factors, such as physical activity [53]. The disparity may also reflect differences in how the economies of these two countries are integrated into the global economy. In rural areas of developing countries, individuals may have less access to low-nutrient, energy-dense products that are high in sugar and fat, and indeed underweight can be more prevalent than overweight/obesity [14,56].

### Household food insecurity

The prevalence of food insecurity among children in Québec (9.4%) was slightly higher than the one reported for the province of Québec through the 2008 Canadian Community Health Survey [30,57]. In 2008, 14.6% of households in the United States reported food insecurity; however, it was not clear how many of these households included children [58]. The present study estimates household food insecurity among Jamaican children to be at a prevalence of 26.4%; to our knowledge, these findings are the first to provide such an estimate. This percentage is disproportionately lower than estimates reported for other developing countries, including an urban population in Caracas, Venezuela (64%) [59], urban and rural samples from Bolivia (70%) [60], and school children 5-12 years old in Bogota, Columbia (76%) [61].

### Association between household food insecurity and overweight/obesity

The present report confirms our hypothesis indicating that in Québec, children who live in food-insecure households have higher odds of being overweight or obese in comparison to children who live in food-secure households. By contrast, in Jamaica food insecurity was associated with decreased odds of being overweight or obese. Similarly, recent studies in Canada and the United States have reported a positive association between food insecurity and childhood obesity [20-22]. However, other studies conducted in similar contexts (e.g., the United States and Mexico) reported negative [23-25] or no relationships between food insecurity and childhood obesity [26,27,62]. As Gundersen and colleagues (2008) suggested, these inconsistencies may be explained by differences in the way food insecurity was measured. For example, in a developing country like Trinidad, Gulliford (2003) showed that food insecurity was associated with an increased likelihood of underweight and not obesity among adults [63]. Unfortunately, studies that might shed light on the relationship between food insecurity and overweight/obesity in children are scarce in developing countries.

One possible explanation for disparities in the relationship between food insecurity and overweight/obesity between developed and developing middle-income countries is that the food-insecurity and overweight/obesity pathways may be different. For example, in developed countries like Canada, social support services available to low-income and food-insecure families (e.g., food subsidies, food stamps, social assistance) have been associated with increased overweight/obesity [64,65]. This may be due to an increased consumption of cheaper and refined carbohydrates, sweetened beverages, high-fat meats, and lower consumption of fruits and vegetables because of monetary constraints. It is known that poorer families may substitute higher-quality foods for cheaper, lower-quality foods and this practice has been associated with overweight and obesity in developed countries [66-68].

By contrast, developing countries like Jamaica have limited social support systems to help with food subsidies. As a result, children from food-insecure and poorer families consume fewer total calories and so are less likely to be overweight or obese. Moreover, the present study's findings may explain why food insecurity and SES share a pathway in relation to overweight and obesity in both countries. Unlike the association between SES and overweight/obesity in Jamaica, the prevalence of overweight/obesity was higher among children from low-SES families and/or children from single-parent families in Québec. In multivariate analyses, SES did not prove to be a significant factor, probably due to the inadequate sample size and its attendant inability to show statistical significance. By contrast, in Jamaica, both variables remained significant, thereby associating high SES with overweight/obesity, whereas food insecurity was associated with reduced odds of overweight/obesity.

### Gender differences

In Québec, when boys and girls were considered separately, food insecurity significantly associated with being overweight or obese only in girls. In children from food-insecure households in Québec, disproportionately more girls were overweight or obese than boys. Additionally, among children who were overweight, more girls than boys (41.9% vs. 27.6%) had single-parent families, and more girls than boys came from low-SES families (34.5% vs. 29.6%). These findings show that the majority of girls and boys from food-insecure households lived in two very different family environments. This difference may explain the gender-specific relationship between household food insecurity and childhood overweight/obesity observed in our study. Moreover, given that, in almost all cases (99%), the head of single-parent families were women in the Québec sample [69], perhaps having an overweight/obese mother exerted a strong influence on girls' body weights since modeling effectively transmits values, beliefs, and practices [70]; however this explanation requires further investigation. When the time and desire to cook are limited among single mothers, they may resort to buying inexpensive, energy-dense, processed foods that have been associated with the obesity epidemic [67].

On the other hand, in Jamaica the prevalence of overweight/obesity was higher among children living in two-parent and blended families, although group differences were not significant at the univariate or multivariate levels. Additionally, boys from higher SES families had greater odds of being overweight/obese than did girls. A positive association between higher SES and overweight/obesity among men, women, and children has been reported in the literature on developing countries [16,17]. Among Jamaican children, the food insecurity-overweight/obesity relationship yielded similar results when boys and girls were analyzed separately. This result has been previously reported for adults in other developing countries [63].

The gender-based differences observed in the present study among children from Québec have been previously reported for adults in other developed countries, such that women in food-insecure households were found to have a higher prevalence of overweight/obesity in comparison to men [45,65,71]. Few studies have examined gender differences in the association between food insecurity and overweight/obesity in children. Two studies found that food-insecure girls were more likely to be overweight than their food-secure counterparts; whereas, among boys, there were no significant findings [22,41]. In developing countries like Jamaica, these previously unstudied emergent differences may well be attributable to the status of the nutritional and epidemiological transition [72,73]. Other mitigating factors, such as family type and SES which our study shows to be present in both developed and developing countries, are consistent with those reported in the literature [16,17,64].

### Strengths and limitations

The most notable strength of this study is that both samples were statistically representative of their populations and both employed standardized weights. The response rate was greater than or equal to 85% in both samples. The Jamaican sample size was adjusted for a 10% refusal rate. The results of the study can thus be generalized to other cases. In addition, objective anthropometric measures of body mass index were obtained.

As our study explores the relationship between food insecurity and overweight/obesity in children from both a developed and a developing country, certain limitations exist. First, given the cross-sectional nature of the data, causality cannot be assigned to the associations observed in this study. Elucidating these relationships will require retrospective longitudinal studies that make it possible to examine temporal sequences of events [41]. Issues relating to the measurement of food insecurity also did not allow us to examine the effects of different levels of food insecurity on overweight/obesity. Furthermore, in the Québec sample, children's level of physical activity was parent-reported rather than measured; thus, children were classified according to subjective information. A number of studies have shown that parents have difficulty accurately estimating and recalling their children's physical activity patterns (e.g., type, intensity, duration, frequency) [34]. Finally, the derivation of the SES variable in the Jamaican sample did not include conventional indicators, such as income and education, as they were not available. This may have resulted in a SES index with reduced discriminatory capacity.

## Conclusions

Food insecurity appears to be positively associated with childhood overweight/obesity in children from the province of Québec, Canada. An inverse relationship is observed among children in Jamaica, a developing country. Gender differences are also apparent in the food insecurity-childhood overweight/obesity association in Québec, such that significantly more girls are found to be "at risk" in comparison to boys. Sex differences in the association between SES, family type, and childhood overweight/obesity are observed in both Québec and Jamaica. The findings of this study suggest that public health interventions which aim to stem the epidemic of overweight/obesity should look beyond biology and individual behaviours to the root causes of the epidemic. Family dynamics, gender differences, and trends in the global food system all influence the spread of overweight/obesity, both in developed and developing countries.

## Abbreviations

SES: socioeconomic status; QLSCD: Québec Longitudinal Study of Child Development; BMI: Body Mass Index; CDC: Centers for Disease Control and Prevention; FAO: Food and Agriculture Organization; USDA: United States Department of Agriculture; FFQ: Food Frequency Questionnaire; IPAQ: International Physical Activity Questionnaire; STATIN: Statistical Institute of Jamaica; OR: Odds Ratios

## Competing interests

The authors declare that they have no competing interests.

## Authors' contributions

LD is the principal investigator and was primarily responsible for the conceptualization of the study. DF, DB, and MG analyzed the data, and DF, DB, and FT wrote the manuscript. GGS, KF, and RW critically revised the manuscript for important intellectual content. All authors read and approved the final manuscript.

## Pre-publication history

The pre-publication history for this paper can be accessed here:

http://www.biomedcentral.com/1471-2458/11/199/prepub
